# Research on China’s adolescent mental health policies — analysis based on PMC index model

**DOI:** 10.3389/fpubh.2024.1408991

**Published:** 2024-08-23

**Authors:** Chengning Yang

**Affiliations:** Shanghai Normal University, Shanghai, China

**Keywords:** adolescent, mental health, mental health education, policy analysis, PMC index model

## Abstract

**Introduction:**

In recent years, the suicide rate among adolescents in China has shown a continuous upward trend, and mental health issues such as depression and excessive anxiety have become increasingly prominent. Awareness and concerns around poor mental health in adolescents is rising among the general public and in academic circles, but there is little research on adolescent mental health policies in China.

**Methods:**

This article reviews the national policies on adolescent mental health from 2000 to 2023, and analyzes and evaluates the selected policy texts through the PMC index model.

**Results:**

The study indicates growing national attention towards adolescent mental health policies in terms of both quantity and quality, with improved policy feasibility and synergy. However, shortcomings exist in the policy formulation process, including a lack of advocacy and supervision-oriented policies, a focus on short to medium-term effects, and inadequate comprehensive planning, hindering their swift implementation.

**Discussion:**

In conclusion, facing the escalating crisis of adolescent mental health, the previous requirements of the education system are no longer sufficient. The government needs to further improve the top-down policy system, weave a safety net for mental health education and preventive intervention, and effectively promote the development of adolescent mental health.

## Introduction

Despite a general decrease in suicide rates over the past two decades, driven by China’s economic development and improvements in the social security system, a worrying transformation has emerged in recent years regarding adolescent suicides, sparking extensive concern across society (1, 2). Particularly, since 2017, a conspicuous upward trend has been observed in the suicide rates among Chinese adolescents (3). Among countries with higher youth suicide rates, Russia, New Zealand, and Japan have all witnessed a decline in suicide rates among adolescents aged 15–19 in recent years (based on the latest data covering the period from 2010 to 2019). Conversely, there has been a slight increase in suicide rates for this age group in countries such as the United States and Australia (4, 5). According to statistics from the National Health Commission, the suicide rates among urban populations of China aged 10–15, 15–20, and 20–25 have increased from 0.96, 1.40, and 1.59 per 100,000 people in 2017 to 1.70, 3.34, and 3.45 in 2021, more than doubling on average. Concurrently, the suicide rates among adolescents in rural areas of China have also reached high levels at 1.66, 3.65, and 3.66 per 100,000 people (3). Research and statistical data from the World Health Organization (WHO) both indicate that suicide is one of the leading causes of death among adolescents (6). Although the suicide rate among Chinese adolescents is not particularly high compared to other major countries, the rapid increase over the past 5 years is a cause for concern.

In addition, adolescent mental health issues and suicidal behaviors among teenagers have also raised widespread concern in society. In April 2024, a 15-year-old junior high school student in Zhejiang committed suicide by jumping from a building due to school bullying. In May, a senior high school student in Henan took his own life by jumping off a building, overwhelmed by exam anxiety. The development of adolescent mental health stands as a significant challenge confronting contemporary Chinese society, necessitating concerted efforts from the government, educational institutions, and the society at large to address. Teenagers are in the stage of receiving school education, and academic pressure is a manifestation that school educators can directly observe. This also leads parents, educators, and even society to believe that academic pressure directly leads to psychological problems such as suicidal behavior. Actually, this viewpoint is very one-sided. Due to their immature psychological development, adolescents often feel uncomfortable due to personal experiences and environmental influences. This negative impact can sometimes lead to mental health issues (7). Especially with the widespread adoption of the Internet, the difficult-to-regulate online environment may give rise to phenomena such as cyberbullying and the shift of school bullying into online forms (8, 9). Education is key to promoting the mental health of adolescents through specialized preventive interventions and the daily educational practices of schools and communities (10).

The mental health education of adolescents is a major social issue faced by countries around the world. In 1894, Alfred Binet of France founded the “Society for the Study of Child Psychology.” This pioneering work marked the beginning of psychology’s application in school education, hence Binet is revered as the “Father of World School Mental Health Education” (11). In 1896, Lightner Witmer founded the first psychological clinic at the University of Pennsylvania, providing psychological services to adolescents struggling academically, thus initiating the application of psychology in education in the United States. Since then, school-based mental health services in the country have evolved into a relatively specialized and standardized system (12). There are a series of policies and regulations to support mental health education for adolescents. As early as 1995, the Joint Committee on National Health Education Standards in the United States issued the “National Health Education Standards,” (13) which established unified national educational goals and implementation standards for mental health education in schools. Various state governments have also enacted laws to incorporate mental health education into the existing curriculum system, along with related suicide prevention, identification, monitoring, and intervention systems (13, 14). The first school counseling room in Japan was established at the University of Tokyo in 1953. It has now been widely popularized in primary, middle, and high schools, and there is a systematic training system and strict assessment system for mental health counselors (16). In the early 1970s, relevant teaching content on mental health was included in the curriculum (16). Compared to countries such as Europe, America, and Japan, China started relatively late in the construction of the adolescent mental health education and education support policy system. However, with the joint issuance of the “National Student Mental Health Work Special Action Plan” by 17 departments including the Ministry of Education and the National Health Commission in 2023, it marks the elevation of strengthening student mental health work to a national strategy”.

This article reviews relevant research on the policy mechanism of adolescent mental health, and quantitatively analyzes and evaluates the policy quality of mental health formulated at the national level in China in the past 20 years. The research results demonstrate the evolution of China’s policy focus on adolescent mental health and the shortcomings of policy mechanisms, providing insights for the development of China’s policy system and related research on adolescent mental health.

## Literature review

Psychologist Erikson proposed the theory of ego identity, which refers to an individual’s integration of self within a specific context, representing the individual’s capacity to seek inner consistency and continuity (17). According to the personality development theory of Erikson, personality development progresses through eight distinct stages, each accompanied by developmental challenges or crises that must be confronted, particularly during adolescence (18). When these challenges are successfully resolved, individuals develop healthy and positive personality characteristics. Conversely, if left unresolved, they can hinder normal personality development, leading to confusion over identity formation during the teenage years (19). Some individuals are able to resolve these conflicts and crises rationally through their own abilities. However, the majority of children and adolescents lack the necessary knowledge and experience to smoothly overcome psychological conflicts (20), thus requiring educational and support interventions from schools, society, and families (21).

Children and adolescents develop not in isolation, but within the interconnected contexts of their families, schools, communities, and broader social environments (22). Recognizing the importance of integrating adolescent mental health and education, much research has focused on school mental health education, and the collaboration between school mental health education and society support, uncovering deficiencies in adolescent mental health. Kodish et al. (23) noted that schools across the United States have implemented suicide prevention protocols to facilitate connections between schools and mental health services. However, significant racial and ethnic disparities in service utilization persist, and considerable differences in perspective exist among school staff, students, and their caregivers regarding these measures. Pearlman et al. (24) demonstrated that while efforts are being made at the national level to enhance mental health education and suicide prevention measures in schools, cumbersome procedures still deter many parents from offering their support. The Whole School, Whole Community, Whole Child (WSCC) model, widely adopted by schools and school districts in recent years, is a comprehensive school health framework that addresses children’s mental health needs within a multi-tiered system of support (25). Schools are universally regarded as vital venues for fostering mental health, with numerous nations and regions adopting a whole school approach to advance mental health education among adolescents (26). Nonetheless, the implementation of this approach is acknowledged as dynamic, intricate, and embedded within a multi-tiered system, necessitating meticulous consideration of the myriad ecological factors that may influence its execution (27).

As the macro impact of mental health and its close relationship with many social factors are gradually being discovered (28, 29), the role of non-mental health professionals, like teachers/educators, in promoting young people’s mental health is increasingly being recognized (30). School-based programs aimed at training young people to support their peers experiencing mental health issues or crises have transitioned from theoretical frameworks to practical implementation (31). Through the collective involvement of non-professionals, more formal/informal educational interventions can be promoted to facilitate the mental health of adolescents through early identification based on schools (32).

In recent years, China’s adolescent mental health issues have attracted increasing attention from society, leading to a growing body of related research. Researchers’ studies can mainly be categorized into two major types: the influencing mechanisms of adolescent mental health and the experiences of international adolescent mental health service systems.

Firstly, research results show that in China, factors such as parent–child relationships (33, 34), parental educational expectations (35), and parents’ socioeconomic status (36) have a significant impact on negative thoughts among adolescents, including depression, suicidal tendencies, and violence. The influence of internet and digital devices on shaping adolescents’ values and causing academic fatigue should also not be ignored (37, 38). Furthermore, a review of studies concerning adolescent mental health services and governance indicates that the primary focus is on countries with more developed policy frameworks for adolescent mental health, such as the United States, Japan, and Canada. These studies delve into analyses of policy transformations in these nations (39), the evolution of institutional mechanisms, and the distinctive features of policy system (40, 41). From the experiences of international adolescent mental health development and governance, the construction of a policy system is particularly crucial (42). However, at present, while there is considerable research and social attention on issues related to China’s adolescent mental health, there is a lack of relevant studies analyzing and evaluating existing policies.

The World Health Organization emphasizes that the development of adolescent mental health requires support from a policy system that involves multi-sectoral collaboration, multi-stakeholder coordination, and multi-link coordination (43). With the development of policy science, policy text quantitative analysis can objectively elucidate key issues such as policy layout, resource allocation, and policy consistency. Policy evaluation constitutes a complex systems engineering endeavor, primarily involving the analysis and judgment of policy operations and design based on specific criteria and procedures. There are myriad methods for policy text evaluation, among which the Policy Modeling Consistency (PMC) index model, proposed by Ruiz Estrada based on the Omnia Mobilis hypothesis, stands out. This model employs traditional text mining methods and mathematical tools for policy quantification assessment (44). The Omnia Mobilis hypothesis perceives all things in the world as interconnected and dynamic, necessitating the consideration of all relevant variables in policy evaluation without limitation on their number and with equal weighting. It aims to analyze the strengths, weaknesses, and internal consistency of policies from multiple dimensions. The core idea of the PMC Index Model is the “*ceteris paribus*” assumption, emphasizing the application of diverse theories, models, and techniques to construct an indicator evaluation system that scrutinizes the internal consistency of any given policy and conducts a comprehensive analysis of its merits and shortcomings (45).

Implementation of the PMC Index Model follows four primary operational steps: First, constructing an evaluation indicator system for policies, categorizing variables, and identifying parameter types; second, building multi-input–output tables, assigning values to policy samples based on this indicator system; third, calculating the PMC index for policy samples; and finally, plotting the PMC surface diagram to visually inspect the scoring situation of various impact factors in policy texts, thereby formulating targeted improvement suggestions based on PMC index scores and areas for refinement. This approach to policy text analysis enables the acquisition of comprehensive raw data, mitigating subjectivity in evaluations and enhancing precision to a certain extent. It is recognized domestically and internationally as a relatively advanced method for policy text evaluation (46, 47). The effectiveness of the PMC Index Model in analyzing policy synergy within China’s public management policy system has been fully validated (48, 49).

Based on the aforementioned content, in order to address the gap in the analysis and evaluation of China’s policy system for adolescent mental health education, this paper conducts text mining based on keywords including adolescent mental health, mental health education and adolescent suicide, gathers relevant policies at the national level in China, and utilizes TF-IDF analysis and the PMC index model to analyze and evaluate selected policy texts, uncovering the development and shortcomings of the existing policy system. The aim is to provide suggestions for improving China’s adolescent mental health policy system and establishing supporting institutional mechanisms.

## Data sources of adolescent mental health policies

This study comprehensively considers the authority, timeliness, and relevance of adolescent mental health policies, following the principles of open and authoritative sample collection. Policy retrieval and collection are conducted through government websites, including the Ministry of Education and the National Health Commission. Using high-frequency terms designated by the World Health Organization for safeguarding adolescent mental health, such as “adolescent mental health,” “mental health in primary and secondary schools,” and “adolescent suicide prevention and control,” as search keywords, policies publicly issued from January 1, 2000, to December 31, 2023, are queried. Policies relating to public consultations, expired or repealed policies, personnel appointments, etc., were excluded, retaining only currently effective policies with specific content. In the end, 10 policy texts were obtained, with details on policy release dates, issuing authorities, and policy titles as shown in Table 1.

**Table 1:** List of policies.

Policy code	Issue date	Issuing authority	Policy title
POL1	16-Mar-02	General Office of the Ministry of Education	Opinions of the Ministry of Education on Strengthening Psychological Health Education for College Students
POL2	12-Jul-02	General Office of the Ministry of Education	Notice from the General Office of the Ministry of Education on Further Strengthening Student Management and Psychological Health Education in Higher Education Institutions
POL3	13-Jan-10	Ministry of Education, Ministry of Health, Central Committee of the Communist Youth League	Opinions on Further Strengthening and Improving Psychological Health Education for College Students
POL4	23-Feb-11	General Office of the Ministry of Education	Basic Construction Standards for Psychological Health Education in Higher Education Institutions (Trial Implementation)
POL5	28-May-11	General Office of the Ministry of Education	Basic Teaching Requirements for Psychological Health Education Courses for College Students
POL6	4-Jul-18	Party Group of the Ministry of Education of the CPC	Guidelines for Psychological Health Education in Higher Education Institutions
POL7	18-Dec-19	National Health Commission and other 12 departments	Healthy China Action - Action Plan for Children and Adolescents’ Mental Health (2019-2022)
POL8	12-Jul-21	General Office of the Ministry of Education	Notice from the General Office of the Ministry of Education on Strengthening Student Psychological Health Management
POL9	27-Apr-23	Ministry of Education and other 17 departments	Comprehensive Special Action Plan for Strengthening and Improving Student Psychological Health Work in the New Era (2023–2025)
POL10	6-Nov-23	General Office of the Ministry of Education	Notice from the General Office of the Ministry of Education on Establishing the National Student Psychological Health Consultation Committee

## Construction and analysis of the PMC index model

Before constructing the PMC index model for evaluating adolescent mental health policies, it is essential to identify all feasible variables. Since the collected 10 policy texts are generally small in size, all within 5,500 words, this study employed TF-IDF analysis to extract keywords from each policy text. The conventional term frequency method is prone to mistakenly identifying routine words lacking informative content as keywords. The advantage of the TF-IDF analysis lies in its use of inverse document frequency methods to calculate word frequency, thereby enhancing the accuracy of keyword extraction. The larger the TF-IDF value, the more important the word. Table 2 presents relevant data on the top 15 keywords from these 10 texts. Changes in keywords reflect the evolving policy focus over time. For instance, earlier policies emphasized psychological counseling and moral education, gradually shifting to emphasize prevention and intervention in addition to counseling. Recent policies also encompass assessment and detection. Furthermore, while the main subjects of earlier policies were schools, teachers, and students, recent policies involve educational authorities, students, parents, and institutions.

**Table 2:** Key policy terms.

POL1		POL2		POL3		POL4		POL5	
Vocabulary	TF-IDF	Vocabulary	TF-IDF	Vocabulary	TF-IDF	Vocabulary	TF-IDF	Vocabulary	TF-IDF
Psychology	0.00910329	Train	0.006443324	Mental health	0.00697951	Psychology	0.00910329	Teacher	0.008856345
Student	0.007693661	Psychology	0.006230883	University student	0.00625973	Student	0.007693661	Student	0.008849364
Counseling	0.006720603	Consulting service	0.006134705	Management work	0.00536548	Consulting service	0.006720603	Crisis	0.008175088
tutor	0.006608584	Tutor	0.005939711	Psychological disorders	0.00357699	Tutor	0.006608584	Psychology	0.007742531
School	0.005335947	Student	0.004908944	Thought	0.00268274	School	0.005335947	Event	0.007644426
Politics	0.004637497	Teacher	0.004497085	Education	0.00268274	Politics	0.004637497	Consulting service	0.006131316
Teacher	0.004637497	Learning	0.003959807	Leadership	0.00268274	Teacher	0.004637497	University student	0.005133203
Issue	0.004066821	Politics	0.003865994	Major	0.00199415	Problem	0.004066821	school	0.005096241
Ideas	0.00405781	Supervision	0.003751512	Event	0.00199415	Thought	0.00405781	Major	0.004586656
Ethical and moral education	0.003558469	Higher education institutions	0.00334043	Information	0.00178849	Train	0.003752766	Full-Time	0.004264431
Ability	0.003478123	Ability	0.003221662	Teaching staff	0.00178849	Quality	0.003752766	Activity	0.004087544
Ideological and moral	0.003334967	Activity	0.003221662	Treat	0.00178849	Moral education work	0.003558469	Education	0.003937246
Quality education	0.003334967	Education	0.003140431	Consulting service	0.00178849	Ability	0.003478123	Mental illness	0.003898636
Society	0.002898436	Guidance	0.003067353	Tutor	0.00178849	Ideology and morality	0.003334967	Train	0.003898636
guidance	0.002898436	Science	0.002761281	prevent	0.00178849	Quality Education	0.003334967	System	0.003406287

POL6		POL7		POL8		POL9		POL10	
Vocabulary	TF-IDF	Vocabulary	TF-IDF	Vocabulary	TF-IDF	Vocabulary	TF-IDF	Vocabulary	TF-IDF
Teaching	0.023975051	Student	0.007257618	Service	0.0059837	Evaluation	0.008575953	Teacher	0.010242246
Psychology	0.021580123	Colleges and universities	0.007236556	Student	0.00565127	Student	0.00741397	Mental health	0.009954917
Mental health	0.020060843	Education	0.007209294	Train	0.00538518	Mental health	0.00717661	Student	0.009663746
University student	0.018284629	Crisis	0.00705178	Education	0.00532146	Train	0.006431965	Education	0.007403719
Skill	0.017276272	Psychology	0.007007356	Community	0.00471203	Education	0.005415812	Evaluation	0.007225086
Curriculum	0.016902199	Mental health	0.006977586	Mechanism	0.00398913	School	0.00453092	Psychology	0.00698717
Classroom	0.012071378	Thought	0.005491194	Children	0.00393326	Parents	0.004348712	Service	0.006437055
Characteristic	0.01096568	Prevent	0.005382943	Youngsters	0.00381757	Problem	0.004348712	Monitor	0.005979231
Teaching method	0.010562456	Politics	0.005382943	Parents	0.00374185	Service	0.004287977	School	0.005931342
Teaching content	0.010562456	Intervene	0.005283278	Health	0.00374185	Psychological counseling	0.004287977	Psychology	0.005170983
Pressure	0.009825324	University student	0.004575995	Psychology	0.00370189	Family	0.004287977	Colleges and universities	0.005092228
Frustration	0.009278821	System	0.004468571	Network	0.00336573	Major	0.00417345	Guidance	0.005092228
Personality	0.009278821	Problem	0.004135175	Environment	0.00336573	Psychology	0.003838396	Children	0.00452461
Psychological counseling	0.008842791	Supervision	0.004037207	Problem	0.00334478	Education department	0.003678055	Problem	0.004442618
Emotion	0.008695272	Quality	0.003203196	Mental health	0.00333988	Instructor	0.00347556	Youngsters	0.004335052

### Determination of variables

Based on the extracted keywords, drawing on the main focus of China’s and international scholars (44, 50)on adolescent mental health policies and existing PMC index analysis research, 9 primary variables and 45 secondary variables were established (Table 3). The 9 primary variables are as follows: nature of the policy (X1); timeliness of the policy (X2); policy domain (X3); issuing authority (X4); incentive measures (X5); level of effectiveness (X6); policy content (X7); policy tools (X8) (51); and policy evaluation (X9).

**Table 3:** Related variables.

Number	Primary variable	Number	Secondary variable	Number	Secondary variable
X_1_	Policy nature	X_1:1_	Publicity initiatives	X_1:2_	Supervision and management
		X_1:3_	Action promotion		
X_2_	Policy timeliness	X_2:1_	Short-term	X_2:2_	Mid-term
		X_2:3_	Long-term		
X_3_	Policy areas	X_3:1_	Educational guidance	X_3:2_	Curriculum construction
		X_3:3_	Supporting services	X_3:4_	Personnel training
X_4_	Policy targets	X_4:1_	Primary and secondary school students	X_4:2_	University student
		X_4:3_	Parents of students	X_4:4_	Education administrators
		X_4:5_	Educators	X_4:6_	Psychological counselors
X_5_	Incentives	X_5:1_	Organizational guidance	X_5:2_	Rewards/Remuneration
		X_5:3_	Supervision and accountability	X_5:4_	Regulatory safeguards
X_6_	Effectiveness level	X_6:1_	Action plan	X_6:2_	Guidelines
		X_6:3_	Implementation standards	X_6:4_	Notification/Opinion
X_7_	Policy content	X_7:1_	Health education	X_7:2_	Psychological counseling
		X_7:3_	Identification detection	X_7:4_	Preventive intervention
X_8_	Policy tools	X_8:1_	Demand	X_8:2_	Supply
		X_8:3_	Environment		
X_9_	Policy evaluation	X_9:1_	Clear goals	X_9:2_	Based on sufficient evidence
		X_9:3_	Detailed planning	X_9:4_	Program Science

### Establishment of secondary variable parameters

The core idea behind the PMC index model for quantitative policy evaluation is to select as many relevant variables as possible and not easily overlook any relevant independent variable. Based on this foundation, when assigning values to relevant items, all secondary variables are weighted equally. Binary parameters are used for setting values, without following the principle of exclusivity. If a policy text contains the content related to a secondary variable, the value for that secondary variable is assigned as 1; otherwise, it is assigned as 0. For example, if a policy not only involves mental health education but also focuses on psychological counseling and effectively promotes the prevention and intervention of mental crises, then X7:1 health education, X7:2 psychological counseling, and X7:4 prevention and intervention should be assigned a value of 1, while X7:3 identification and detection should be assigned a value of 0 since the policy does not emphasize this aspect.

### Establishment of multi-input–output table

By quantifying single variables from multiple perspectives using the above method, a multi-input–output table for policy texts is constructed by combining the 9 primary variables and 45 secondary variables. This table serves as a data analysis framework for the subsequent calculation of the PMC index model. Specific results are shown in Table 4.

**Table 4:** Multi input–output table.

Variables		Policy code									
Primary variable	Secondary variable	1	2	3	4	5	6	7	8	9	10
X1	X1.1	0	0	0	0	0	0	0	0	0	1
	X1.2	0	0	1	0	0	0	0	1	1	1
	X1.3	1	1	1	1	1	1	1	1	1	1
X2	X2.1	1	0	1	1	1	1	0	1	1	1
	X2.2	1	1	1	1	1	1	1	1	1	1
	X2.3	0	1	0	0	0	0	1	0	0	0
X3	X3.1	1	1	1	1	0	1	1	1	1	1
	X3.2	1	1	0	0	1	1	1	0	1	1
	X3.3	0	0	0	0	1	1	1	1	1	1
	X3.4	1	1	0	1	1	1	0	0	1	1
X4	X4.1	0	0	0	0	0	0	0	1	1	1
	X4.2	1	1	1	1	1	1	1	1	1	1
	X4.3	0	0	0	0	0	0	0	1	1	1
	X4.4	1	1	1	1	1	0	1	1	1	1
	X4.5	1	1	1	1	1	1	1	1	1	1
	X4.6	1	1	1	1	1	1	1	1	1	1
X5	X5.1	1	1	1	1	1	1	1	1	1	1
	X5.2	1	1	0	1	1	0	0	1	1	1
	X5.3	0	0	0	0	1	0	1	1	0	1
	X5.4	0	0	0	0	0	0	0	0	0	1
X6	X6.1	0	0	0	0	0	0	0	1	0	1
	X6.2	0	1	0	0	0	0	1	0	0	0
	X6.3	0	0	0	0	1	1	0	0	0	0
	X6.4	1	0	1	1	1	1	1	1	1	1
X7	X7.1	1	1	1	1	1	1	1	1	1	1
	X7.2	1	1	1	1	1	1	1	1	1	1
	X7.3	0	0	0	0	1	0	1	1	1	1
	X7.4	0	0	0	0	1	0	1	1	1	1
X8	X8.1	0	0	0	0	0	0	1	1	1	1
	X8.2	1	1	1	1	1	1	1	1	1	1
	X8.3	1	1	1	1	1	1	1	1	1	1
X9	X9.1	1	1	1	1	1	1	1	1	1	1
	X9.2	0	0	0	0	0	1	0	0	1	0
	X9.3	0	0	0	0	0	0	0	0	0	0
	X9.4	0	1	0	0	1	1	1	1	1	1

### Policy quantitative evaluation

Following the calculation method of the PMC index and based on the variable parameters in the multi-input–output table, the comprehensive scores of adolescent mental health policies are calculated according to Equation 1. Where i represents the primary index and j represents the secondary index. The calculation method for the PMC index value of each policy is as follows:


$$\overset{9}{\underset{i=1}{\sum }}X_{i}\left[\overset{x}{\underset{j=1}{\sum }}\frac{X_{ij}}{T\;\left(X_{ij}\right)}\right]$$

(1) $$\begin{array}{l} \mathrm{PMC}=X_{1}\left[\overset{3}{\underset{j=1}{\sum }}\frac{X_{1j}}{3}\right]+X_{2}\left[\overset{3}{\underset{j=1}{\sum }}\frac{X_{2j}}{3}\right]+X_{3}\left[\overset{4}{\underset{j=1}{\sum }}\frac{X_{3j}}{4}\right]\\ +X_{4}\left[\overset{6}{\underset{j=1}{\sum }}\frac{X_{4j}}{6}\right]+X_{5}\left[\overset{4}{\underset{j=1}{\sum }}\frac{X_{5j}}{4}\right]+X_{6}\left[\overset{4}{\underset{j=1}{\sum }}\frac{X_{6j}}{4}\right]\\ +X_{7}\left[\overset{4}{\underset{j=1}{\sum }}\frac{X_{7j}}{4}\right]+X_{8}\left[\overset{3}{\underset{j=1}{\sum }}\frac{X_{8j}}{3}\right]+X_{9}\left[\overset{4}{\underset{j=1}{\sum }}\frac{X_{9j}}{4}\right] \end{array}$$

Since there are 9 primary indices selected, the PMC index ranges from 0 to 9. According to the evaluation criteria proposed by Estrada (44), specific values calculated by the PMC index are divided into different levels: when the PMC index ranges between 8 and 9.0, the policy coherence is highest and categorized as “perfect”; a score between 6 and 7.99 is considered “good”; a score between 4 and 5.99 is categorized as “acceptable”; and if the score falls between 0 and 3.99, the policy coherence is weak and classified as “poor.”

From the policy evaluation results (Table 5), it can be seen that the latest policy issued in 2023 (POL10) ranks first in the PMC index, but it has not reached the “perfect” level (8.0–9.0). The evaluation results of the 7 policies issued before 2019 (POL1-POL7) are rated as “acceptable,” with policy POL3 having the lowest PMC index of 4.17, especially in the areas of policy domain (X3), policy target (X4), and policy evaluation (X9) where the scores of primary variables are relatively low. The 3 policies issued after 2019 all scored above 6, classified as “good.” The overall excellence rate is 33.33%, slightly lower. Policies issued after 2011 all scored above 5 and show an overall upward trend, indicating an increasing emphasis on adolescent mental health development by the country, with policy formulation focusing more on scientificity and coherence.

**Table 5:** Policy evaluation.

Policy code	PMC index	Evaluation results
POL 1	4.58	Acceptable
POL 2	4.83	Acceptable
POL 3	4.17	Acceptable
POL 4	4.33	Acceptable
POL 5	5.83	Acceptable
POL 6	5.17	Acceptable
POL 7	5.92	Acceptable
POL 8	6.58	Good
POL 9	6.83	Good
POL 10	7.67	Good

### Construction of PMC surface

**Figure 1:**
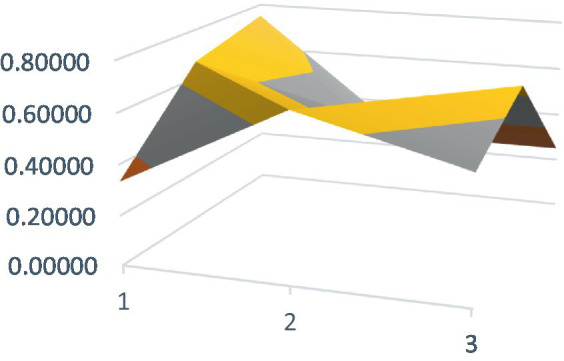
PMC surface diagram of POL1.

**Figure 2:**
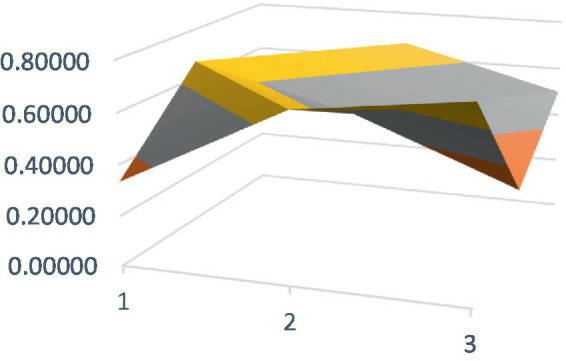
PMC surface diagram of POL2.

**Figure 3:**
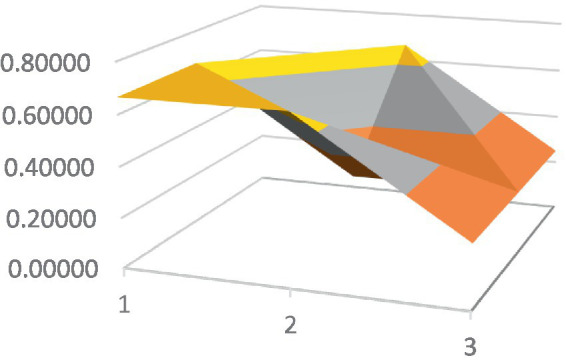
PMC surface diagram of POL3.

**Figure 4:**
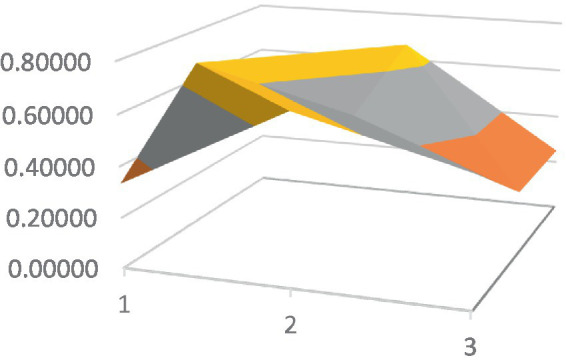
PMC surface diagram of POL4.

**Figure 5:**
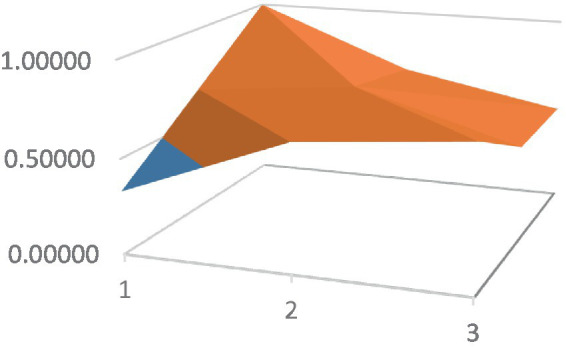
PMC surface diagram of POL5.

**Figure 6:**
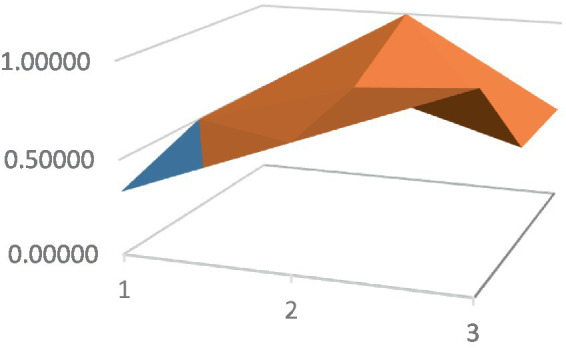
PMC surface diagram of POL6.

**Figure 7:**
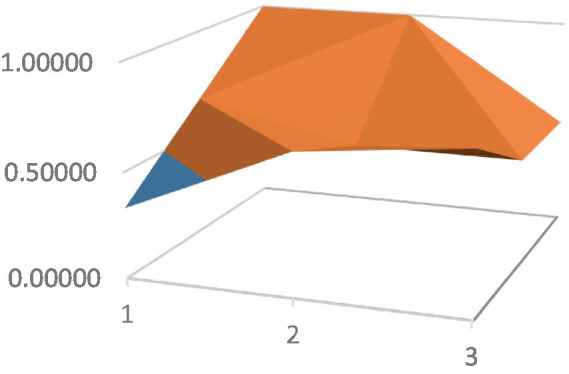
PMC surface diagram of POL7.

**Figure 8:**
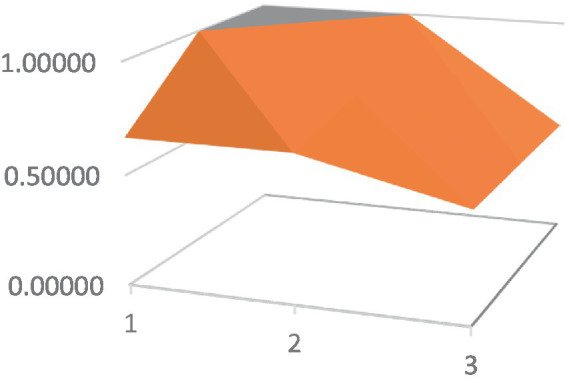
PMC surface diagram of POL8.

**Figure 9:**
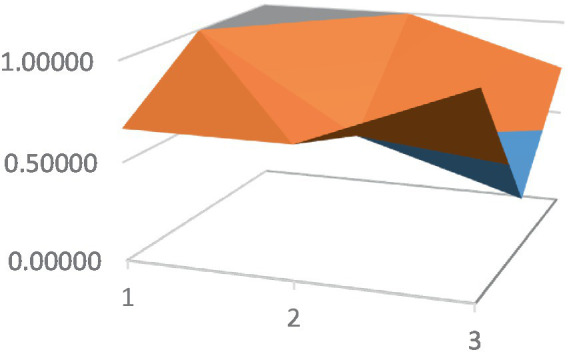
PMC surface diagram of POL9.

**Figure 10:**
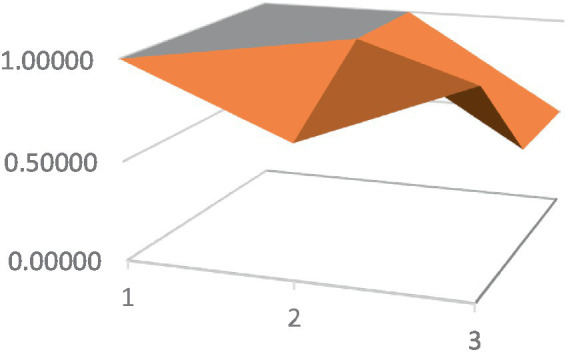
PMC surface diagram of POL10.

Constructing PMC surfaces can visually display the calculated PMC index in a more vivid and intuitive manner, providing a visual analysis of the strengths and weaknesses of policies from multiple dimensions. The PMC surface is a three-dimensional solid surface with symmetry and balance. Firstly, based on the PMC index of the 9 primary variables for each policy, a 3×3 matrix is constructed as shown in Equation 2. Subsequently, using Matlab for plotting, PMC surfaces for each policy are constructed (Figures 1–10), allowing for an intuitive judgment of the strengths and weaknesses of various dimensions of a certain adolescent mental health policy through the undulations of the surface, enabling a scientific, macroscopic, and clear assessment of the overall level of the policy.


(2) $$\left(\begin{array}{l} X_{1}\left[\overset{3}{\underset{j=1}{\sum }}\frac{X_{1j}}{3}\right]X_{2}\left[\overset{3}{\underset{j=1}{\sum }}\frac{X_{2j}}{3}\right]X_{3}\left[\overset{4}{\underset{j=1}{\sum }}\frac{X_{3j}}{4}\right]\\ X_{4}\left[\overset{6}{\underset{j=1}{\sum }}\frac{X_{4j}}{6}\right]X_{5}\left[\overset{4}{\underset{j=1}{\sum }}\frac{X_{5j}}{4}\right]X_{6}\left[\overset{4}{\underset{j=1}{\sum }}\frac{X_{6j}}{4}\right]\\ X_{7}\left[\overset{4}{\underset{j=1}{\sum }}\frac{X_{7j}}{4}\right]X_{8}\left[\overset{3}{\underset{j=1}{\sum }}\frac{X_{8j}}{3}\right]X_{9}\left[\overset{4}{\underset{j=1}{\sum }}\frac{X_{9j}}{4}\right] \end{array}\right)$$

By calculating the PMC index of the above 10 adolescent mental health policies and plotting the PMC curves, the status of each primary index of these 10 policies can be visually represented through Figures 1–10. From the PMC surface diagram, it can be seen that the distribution of various dimensions of the previous policy (POL1-POL4) is uneven, but the quality is generally low. The overall quality of follow-up policies (POL5-POL6) has improved, but there are a few dimensions that are extremely low, which has affected the overall effect. In the later stage of policy (POL7-POL10), while improving the overall quality, more emphasis is placed on the mutual coordination of various dimensions, especially POL8-POL10.

## Discussion

Observing horizontally across Table 4 reveals a notable differentiation in the overall trend of evaluation indicator scores. Significant disparities are particularly evident in areas such as “policy targets” (X4), “incentive measures” (X5), “policy content” (X7), and “policy instruments” (X8). With the progression of years, there has been an overall trend of improvement in the scores of the majority of primary variables. Specifically, an increasing number of secondary variables are receiving a score of 1, indicating that policies now more frequently incorporate content relevant to these indicators or meet the requirements outlined by them.

Combining Tables 3, 4, it can be seen that in the early stage, “policy targets” (X4) were mostly concentrated on “college students” (X4.2), “education managers” (X4.4), “educators” (X4.5), and “psychological counselors” (X4.6). In recent years, there has been an increase in reported cases of suicide among primary and secondary school students, which has aroused widespread social concern, also indicating a trend toward younger age groups in the manifestation of mental health issues. The policy targets introduced after 2021 have broadened from focusing solely on college students to encompass “primary and secondary school students” (X4.1), and more emphasis has been placed on the collaboration between families and schools (X4.3) and the participation of multiple stakeholders. “Incentive” (X5) measures have gradually increased from unilateral “organization and guidance” (X5.1) to “rewards/remuneration” (X5.2) and other methods. The evolution of “policy content” (X7) is even more prominent. In the policies issued earlier, the main focus was on “health education” (X7.1) and “psychological counseling” (X7.2). As the scientific understanding and practices in adolescent mental health have advanced, later policies have increasingly emphasized identification and assessment (X7.3) along with “preventive interventions” (X7.4) as new priorities. “Policy tools” (X8) have also diversified from focusing only on “supply tools” (X8.2) such as infrastructure construction and talent cultivation and “environmental tools” (X8.3) such as standardized management and organizational construction to “demand tools” (X8.1) such as demonstration and resource sharing.

However, as illustrated in Table 4, the overall scores of primary variables such as “policy nature” (X1), “policy effectiveness” (X2), “effectiveness level” (X6), and “policy evaluation” (X9) in terms of evaluation indicators are less than ideal. This is mainly reflected in an uneven distribution of evaluation indicator scores among the secondary variables, with some indicators showing a lack of scoring. For example, in the nature of policies (X1), there are more action promoting policies (X1.3), and fewer “publicity initiatives” (X1.1) and “supervision management” (X1.2). In terms of “policy timeliness” (X2), the coverage of policy impact effectiveness is more in the medium and short term (X2.1, X2.2), and less in the “long term” (X2.3). There are more “notification/opinions” (X6.4) in the “effectiveness level” (X6), but fewer “action plans” (X6.1), “guidelines” (X6.2), and “implementation standards” (X6.3). However, there are more “policy evaluation” (X9) that align with “clear goals” (X9.1) and” program science” (X9.4), while there are fewer that align with sufficient basis (X9.2) and “detailed planning” (X9.3).

In particular, there are individual secondary variables that are rarely covered by policies, such as “regulatory safeguards” (X5.4) type incentives in “incentive” (X5) measures; the evaluation in “policy evaluation” (X9) that conforms to the “detailed planning” (X9.3).

Based on these findings, several recommendations are proposed: Firstly, the nature of policies should focus on targeted actions while also considering creating a conducive environment for broad societal participation. Relying solely on schools for adolescent mental health education is a misconception, as numerous China’s and international studies show that the causes of adolescent mental health issues go beyond academic pressure and insufficient mental health education. The development of good/positive adolescent mental health requires long-term identification, monitoring, and intervention, involving various government departments such as health and education, along with educators, families, and social entities of different age groups. It is essential to establish a collaborative system involving government agencies, society, schools, families, and healthcare institutions to create a supportive environment and monitoring mechanism for healthy development. Secondly, legislation should be enacted to protect adolescent mental health. Currently, the lack of specific indicators and legal safeguards to achieve overall goals in the psychological health policy system for primary and secondary schools is apparent. The 2022 “World Mental Health Report” calls for the formulation and implementation of policies and laws aimed at promoting and protecting mental health. “Over 40 countries have developed suicide prevention strategies, with more than 20 countries incorporating adolescent mental health into their national health plans.” It is essential for the state to legislate and establish corresponding regulations and policies, clearly defining the responsibilities of government departments, society, and schools regarding adolescent mental health, and providing clear definitions. Although China has issued documents such as the “Healthy China Action - Children and Adolescents Mental Health Action Plan (2019-2022),” the “14th Five-Year National Health Plan,” (52) and the “National Mental Health Work Plan (2015–2020),” (53) emphasizing the importance of mental health promotion and service system construction, there is still a lack of highly standardized and operational legislation on adolescent anti-suicide and specific indicators for adolescent mental health. Thirdly, provide cross-departmental coordination rules, implement measures to strengthen responsibilities, and establish specific incentive mechanisms. The primary challenge in promoting good adolescent mental health development is the dispersed functions of government, schools, families, healthcare institutions, and social organizations, lacking coordination and synergy. Appropriate top-level design is indispensable. Currently, various health departments in China have issued multiple mental health-related plans, but none have explicitly mentioned or emphasized the implementation measures of specific issues such as adolescent suicide prevention and governance. It is crucial to clarify the division of responsibilities among various levels, entities, and departments, and promote the hard connectivity of basic infrastructure for adolescent mental health development and the soft integration of coordinated mechanisms.

## Conclusion and recommendations

This study focuses on the analysis of 10 adolescent mental health policies enacted between 2000 and 2023, constructing a PMC index evaluation model for quantitative analysis of policy texts. The research results indicate that both in terms of quantity and quality of policies, the national attention to adolescent mental health development is increasing, with policy feasibility and synergy overall improving. However, there are aspects overlooked in the policy formulation process, such as the lack of advocacy-oriented and supervision management-oriented policies in creating a supportive environment and establishing monitoring mechanisms for adolescent mental health. The policy timeliness mostly focuses on short to medium-term effects, lacking guiding outlines and implementation standards with long-term impacts, particularly lacking legislative safeguards. Moreover, there is a lack of comprehensive planning, hindering the rapid implementation of policies.

In conclusion, facing the escalating crisis of poor adolescent mental health, the previous requirements of the education system are no longer sufficient. It requires the broad participation of all stakeholders in society to create an atmosphere of cherishing life and valuing well-being, as well as a supportive environment, ultimately reducing suicide rates by intervening in the causes and mediators of negative impacts on good adolescent mental health. In this process, the driving force of policy flow should not be underestimated. The government needs to further improve the top-down policy system, weave a safety net for mental health education and preventive intervention, and effectively promote the development of adolescent mental health.

Due to the large number of local administrative units in China and the obvious imbalance in educational resources, this article only evaluates policies at the national level. Therefore, it may have overlooked the detailed and scientific policy layout of some regional governments in the field of adolescent mental health education. In the future, we plan to sort out and evaluate the policies on adolescent mental health education issued by smaller administrative units, in order to draw conclusions and suggestions that are more conducive to policy makers.

## Data Availability

The raw data supporting the conclusions of this article will be made available by the authors, without undue reservation.
